# Profiling the Adrenergic System in Breast Cancer and the Development of Metastasis

**DOI:** 10.3390/cancers14225518

**Published:** 2022-11-10

**Authors:** Daniela M. Sousa, Veronica Fernandes, Catarina Lourenço, Carina Carvalho-Maia, Helena Estevão-Pereira, João Lobo, Mariana Cantante, Marina Couto, Francisco Conceição, Carmen Jerónimo, Luisa Pereira, Meriem Lamghari

**Affiliations:** 1 i3S—Instituto de Investigação e Inovação em Saúde, Universidade do Porto, 4200-135 Porto, Portugal; 2 INEB—Instituto Nacional de Engenharia Biomédica, Universidade do Porto, 4200-135 Porto, Portugal; 3 IPATIMUP—Instituto de Patologia e Imunologia Molecular da Universidade do Porto, 4200-135 Porto, Portugal; 4 Cancer Biology and Epigenetics Group, Research Center of IPO Porto (CI-IPOP)/RISE@CI-IPOP (Health Research Network), Portuguese Oncology Institute of Porto (IPO Porto)/Porto Comprehensive Cancer Center Raquel Seruca (Porto.CCC), 4200-072 Porto, Portugal; 5 Department of Pathology, Portuguese Oncology Institute of Porto (IPO Porto), 4200-072 Porto, Portugal; 6 ICBAS—Instituto de Ciências Biomédicas Abel Salazar, Universidade do Porto, 4050-313 Porto, Portugal

**Keywords:** adrenoreceptors, breast cancer, bone metastasis, stress, sympathetic nervous system

## Abstract

**Simple Summary:**

A cancer diagnosis can be a highly stressful experience and breast cancer (BC) patients are at a higher risk of suffering from depression and chronic stress. Thus, the activation of the adrenergic system might negatively impact the disease’s progression. In this study, we propose to perform a comprehensive study on the adrenergic profile in BC and correlate it with the occurrence of metastasis. Our analysis showed that BC patients are indeed under the control of the sympathetic nervous system (SNS), since the circulating levels of catecholamines are elevated in BC patients at all stages of the disease. Metastatic bone biopsies express sympathetic nerve fibers and adrenoreceptors. Moreover, we also observed a pronounced gene expression and the downregulation of adrenoreceptors and catecholamine metabolic enzymes in BC tissues. This downregulation appears to be detrimental for the prognosis of the disease. The evidence gathered will be crucial in the design of new therapeutic approaches targeting SNS in BC.

**Abstract:**

Epidemiological studies and preclinical models suggest that chronic stress might accelerate breast cancer (BC) growth and the development of metastasis via sympathetic neural mechanisms. Nevertheless, the role of each adrenergic pathway (α1, α2, and β) in human samples remains poorly depicted. Herein, we propose to characterize the profile of the sympathetic system (e.g., release of catecholamines, expression of catecholamine metabolic enzymes and adrenoreceptors) in BC patients, and ascertain its relevance in the development of distant metastasis. Our results demonstrated that BC patients exhibited increased plasma levels of catecholamines when compared with healthy donors, and this increase was more evident in BC patients with distant metastasis. Our analysis using the BC-TCGA database revealed that the genes coding the most expressed adrenoreceptors in breast tissues (*ADRA2A*, *ADRA2C,* and *ADRB2,* by order of expression) as well as the catecholamine synthesizing (*PNMT*) and degrading enzyme (*MAO-A* and *MAO-B*) genes were downregulated in BC tissues. Importantly, the expression of *ADRA2A*, *ADRA2C,* and *ADRB2* was correlated with metastatic BC and BC subtypes, and thus the prognosis of the disease. Overall, we gathered evidence that under stressful conditions, both the α2- and β2-signaling pathways might work on a synergetic matter, thus paving the way for the development of new therapeutic approaches.

## 1. Introduction

In 2021, breast cancer (BC) was recognized as the most commonly diagnosed type of cancer, representing around 25% of all new cancer cases diagnosed in females worldwide [1,2]. With the implementation of earlier diagnosis and the improvement in adjuvant therapies, the patient mortality rate has slowly been decreasing in developed countries [3,4,5,6]. The shortcoming occurs when disseminated BC reaches distant organs such as the bone, lungs, liver, and brain [7], leading to the development of metastatic BC. Indeed, metastatic BC affects 6–10% of women at the time of diagnosis, which translates, on a 5-year overall survival rate, of only 27% [6,8,9]. The clinical management of metastatic BC is still challenging, since there are no effective therapeutic approaches to surpass and solve the progression of the disease.

Cumulative evidence suggests a striking link between the sympathetic nervous system (SNS) and BC [10,11]. In a point of fact, a sprouting of sympathetic tyrosine hydroxylase (TH)-positive nerve fibers within primary BC surrounding tissue was associated with a poor clinical prognosis [12]. Additionally, epidemiological studies and in vivo animal models revealed that stress, and the concomitant sympathetic hyperactivation, accelerated BC growth and metastasis via sympathetic neural mechanisms [13,14,15], thus suggesting that sympathetic hyperactivity has the potential to negatively affect the progression of the disease.

In response to a specific stimuli (e.g., anxiety, depression, stress, poor coping, social isolation), there is a “fight-or-flight” response triggered by the SNS that translates into the release of norepinephrine (NE) and epinephrine (EPI), also known as catecholamines. The actions of the SNS are regulated locally through the release of NE by the sympathetic nervous terminals that directly innervate the target organs, or systemically through the systemic release of NE and EPI (in a proportion of 20:80, respectively) by the adrenal glands into circulation [16]. Importantly, in chronic stress conditions, the physiological systems are, besides catecholamines, exposed to glucocorticoids (e.g., cortisol (CORT)), released into circulation from the adrenal glands upon the activation of the hypothalamic–pituitary–adrenal (HPA) [17].

The sympathetic neuromediators, NE and EPI, can bind to nine different adrenergic receptors (ADR): α1-ADR (α1a, α1b, and α1d), α2-ADR (α2a, α2b, and α2c) and β-ADR (β1, β2, β3) [18,19]. For this reason, the ADRs not only show distinct patterns of tissue distribution, but can also develop diverse functions and sometimes opposite actions, ultimately regulating a panoply of physiological processes. In the context of BC, α2a- and β2-ADR have been described to be involved in BC. In fact, β2 was the first ADR described to be expressed in breast tissues [20] and BC cell lines [21], and the ADR mostly studied in this field. Several studies have demonstrated that β2-stimulation promotes tumor growth using BC cell lines [22,23,24,25,26], which is in accordance with its stimulatory G-protein nature. However, other studies have described the opposite effect on tumor cell proliferation [27,28], and thus the role of β2-activation on BC proliferation is still controversial. Aside from proliferation, β2-ADR has also been associated with increased BC cell migration [29,30,31,32,33] and metastasis [26,30,34,35,36]. Additionally, some reports have provided evidence for a direct role of the β-adrenergic signaling pathway in the acceleration of tumor angiogenesis [37,38]. Additionally, apart from the β-adrenergic signaling pathway, there is a lack of reports addressing the role of other adrenergic pathways in BC and metastasis.

In general, these reports support the hypothesis that chronic SNS activation plays a critical role in the development of BC and on the establishment of metastatic BC, specifically to the bone [34]. Nevertheless, the link between stress-related signaling pathways and BC metastasis, and the mechanisms behind them, remain poorly understood. Given the high heterogeneity of the adrenergic signaling, it is still not clear what the adrenergic profile of the BC tissues is and its correlation with the BC subtype and prognosis. Specifically, how this adrenergic profile can influence the BC development of chronically stressed patients.

Based in this knowledge and as a starting point, herein, we propose to perform a comprehensive characterization on the profile of the adrenergic system (e.g., release of catecholamines, expression of catecholamine metabolic enzymes, and adrenergic receptors) in BC, and ascertain its relevance in the development of distant metastasis, specifically to the bone—the most common site in metastatic BC [39]. The correlations established here will be further explored in upcoming studies, thus paving the way for the development of more effective treatments.

## 2. Materials and Methods

### 2.1. Human Samples

Plasma samples retrieved from female heathy donors (n = 18) and female BC patients (n = 72) as well as bone biopsies retrieved from female BC patients (n = 44) were included in this study. All samples were collected at the Portuguese Oncology Institute of Porto (IPO PORTO) and informed consent was obtained from all individual participants. The clinicopathological data relevant for this study were extracted from the patients’ charts and revised by experienced pathologists.

### 2.2. Quantitative ELISA Analysis

Peripheral blood was collected from BC patients into EDTA-containing tubes and centrifuged at 2000 rpm for 10 min at 4 °C. Plasma was immediately separated, aliquoted into 1.5 mL tubes, and properly stored at −80 °C. The levels of NE, EPI, and CORT present in the plasma samples were quantified using commercially available enzyme immunoassay kits: Epinephrine/Norepinephrine ELISA Kit (Abnova, Walnut, CA, USA), and Cortisol Competitive Human ELISA Kit (Invitrogen, Carlsbad, CA, USA), following the manufacturer’s guidelines.

### 2.3. Immunofluorescence Analysis

Formalin-fixed paraffin-embedded 4 μm thickness sections obtained from the bone biopsies of BC patients were submitted to immunofluorescence staining against sympathetic markers (TH, α2a, and β2). Briefly, tissue sections were deparaffinized, rehydrated, and submitted to antigen retrieval by incubating samples at 98 °C in a 10 mM citrate buffer (pH 6.0). After quenching the inherent endogenous fluorescence with 0.1% NaBH_4_ and 100 mM NH_4_Cl, sections were incubated with blocking buffer (10% FBS, 1% BSA, 0.2% Triton X-100) for 1 h at room temperature. Primary antibodies: (i) anti-TH (dilution 1:100, Millipore, Temecula, CA, USA); (ii) anti-α2a (dilution 1:200, Abcam, Cambridge, MA, USA); (iii) anti-β2 (dilution 1:100, Proteintech, USA); or (iv) blocking buffer (negative control) were applied overnight at 4 °C. For signal detection, tissue sections were incubated for 1 h at room temperature with a secondary antibody anti-rabbit Alexa Fluor 568 antibody (dilution 1:1000, Life Technologies, Carlsbad, CA, USA), incubated with DAPI, and then mounted with the Fluoroshield Mounting Medium (Abcam, Cambridge, MA, USA). The TH, α2a, and β2 labelling specificities were confirmed using human specimens from the previously published work of our laboratory [40,41]. Immunostaining images were acquired and analyzed using a confocal laser scanning microscope (Leica TCS SP5 microscope, Leica Microsystems, Wetzlar, Germany).

### 2.4. Transcriptomic Analysis

#### 2.4.1. Human BC Tissue Samples Dataset

We used the Cancer Genome Atlas (TCGA; https://cancergenome.nih.gov/; accessed on 5 November 2021) to collect the gene expression data of 1123 BC tissue samples and 99 adjacent normal counterparts (TCGA-BC). The expression of genes was quantified as FPKM (fragments per kilo base of transcript per million mapped fragments), which were provided by the TCGA consortium. Clinical and somatic mutation metadata were also provided by the TCGA consortium at https://gdc.cancer.gov/, accessed on 5 November 2021.

#### 2.4.2. Human BC Cell Lines Dataset

We used the public Cancer Cell Line Encyclopedia database (CCLE; https://sites.broadinstitute.org/ccle/; accessed on 22 March 2022) to collect the gene expression profiles of the BC cell lines. We excluded cell lines lacking metadata as well as cell lines whose status of primary markers and the BC subtype are still not clearly identified [42], resulting in a final number of 52 human BC cell lines analyzed.

### 2.5. Statistical Analysis

Normal distribution was evaluated through the Shapiro–Wilk normality test. Depending on Gaussian distribution, differences between groups were assessed by non-parametric or parametric tests. We performed the Wilcoxon test for a comparison between the TCGA-BC tumor samples and their adjacent normal counterparts by the TCGA-BC subtype and stage. Correlations were tested with the Pearson’s correlation test. Statistical significance was accepted when the *p*-value < 0.05. Outliers were removed. All statistical analyses were performed using R software (version 4.2.1; San Francisco, CA, USA) and graphs were built using GraphPad Prism software for macOS (version 9.4.0; San Diego, CA, USA). The overall survival curves were performed in R using the Kaplan–Meier method.

## 3. Results

### 3.1. BC Patients with Bone Metastasis Exhibit Elevated Levels of Circulating EPI

**Figure 1:**
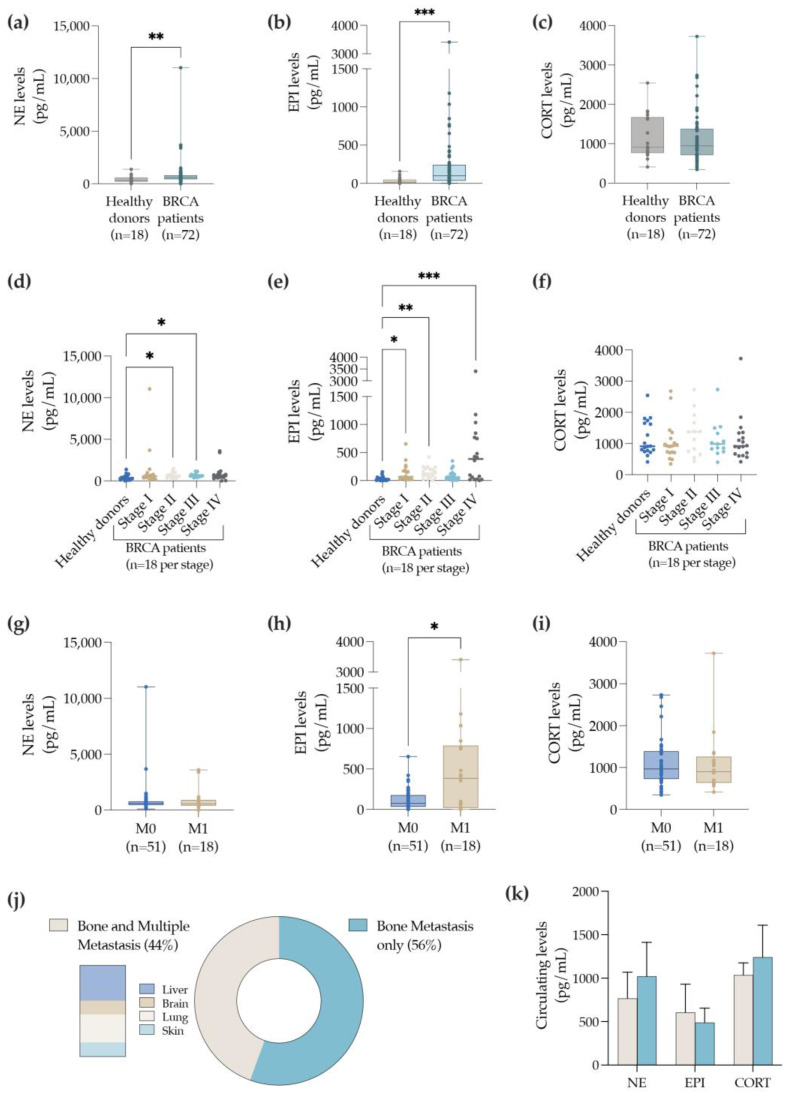
Plasma levels of catecholamines in a cohort of BC patients. Systemic levels of NE (**a**) and EPI (**b**) CORT (**c**) in the BC patients (n = 72) versus healthy donors (n = 18). The circulating levels of NE (**d**), EPI (**e**) and CORT (**f**) at all stages of the disease were discriminated in relation to the healthy donors (n = 18 per stage). Additionally, the systemic levels of NE (**g**), EPI (**h**), and CORT (**i**) in the BC patients displaying no distant metastasis (M0; n = 51) versus the BC patients with distant metastasis (M1; n = 18) were also analyzed. BC patients with distant metastasis exhibiting bone metastasis only (56%) or multiple metastasis sites (44%), namely, in bone, liver, brain, lung, and skin are shown (**j**). The systemic levels of catecholamines, NE and EPI, and CORT were compared between BC patients displaying bone metastasis only versus multiple metastasis sites (**k**). *p*-value < 0.05 (*); <0.01 (**), <0.001 (***) was considered statistically significant.

In acute and chronic stress conditions, the SNS triggers the release into the circulation of catecholamines (NE and EPI) or/and CORT, by the adrenal glands [16,17]. As such, we propose analyzing the levels of circulating markers of sympathetic hyperactivity (NE, EPI, and CORT) in the BC patients. To perform this analysis, a cohort of BC patients comprising all stages (I, II, III, and IV) of BC disease was selected (for clinicopathological details please refer to Table 1) and the plasma levels of NE, EPI, and CORT were quantified by ELISA (Figure 1).

Our analysis demonstrated that the plasma levels of both NE (Figure 1a) and EPI (Figure 1b) were elevated in BC patients when compared to healthy donors. Furthermore, there were no differences in the circulating CORT levels (Figure 1c). Importantly, when compared to the levels of healthy donors, NE was differentially elevated in BC patients at stage II and III of the disease (Figure 1d), while the circulating levels of EPI were significantly higher at early stages (stage I and stage II) and in an advanced stage (stage IV) of the disease (Figure 1e). In accordance with previous results (Figure 1c), no differences in the CORT levels were observed between stages when compared to the healthy donors (Figure 1f).

BC prognosis decreases considerably when distant metastasis occurs [1]. Additionally, it is described that the expression of stressor markers might have an impact on the progression of the disease [11,12]. Thus, to empower our analysis, the levels of sympathetic markers were also evaluated in BC patients with (M1; n = 18) and without distant metastasis (M0; n = 51). In the case of the NE levels, there were no differences between the M0 and M1 patients (Figure 1g). Interestingly, BC patients with distant metastasis (M1) displayed higher levels of circulating EPI when compared with BC patients with no distant metastasis (Figure 1h). Moreover, the biochemical levels of the stress marker CORT were again not different (Figure 1i).

Considering that bone is a common site for BC metastasis [43], and that all cases of patients with distant metastasis M1 (n = 18) exhibited bone metastasis, the levels of the sympathetic markers previously analyzed (NE, EPI, and CORT) were then explored between the patients with bone metastasis only (56% of the cases) versus the patients with multiple sites of metastasis (44% of the cases) including the bone, liver, brain, lung, and skin (Figure 1j). Nonetheless, no differences were observed when comparing the M1 BC patients with bone metastasis only, with M1 patients carrying multiple sites of metastasis (Figure 1k). Overall, these results demonstrate that the levels of circulating catecholamines (e.g., NE and EPI) were elevated in the cohort of BC patients explored in this study. In particular, EPI was augmented at both early and advanced stages of the BC, predominantly when distant metastasis occurred, confirming a possible correlation with the progression of BC disease.

### 3.2. Bone Metastasis Biopsies Retrieved from BC Patients Express Sympathetic Nerve Fibers and the Adrenergic Receptors—α2a and β2

**Figure 2:**
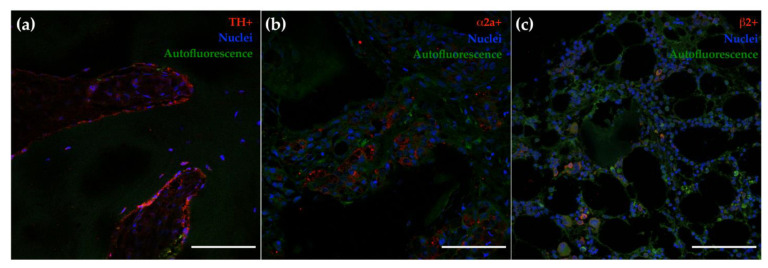
Representative images of immunofluorescence analysis on bone biopsies retrieved from BC patients. TH-positive (**a**), α2a-positive (**b**), and β2-positive (**c**) staining. Tissue autofluorescence is denoted in green. Scale bar = 100 µm.

Bone is a predominant site for BC cell dissemination [44]. When bone metastasis occurs, the BC patients’ survival rate decreases considerably [6,9]. Since BC patients with bone metastasis (M1 patients) exhibit higher levels of circulating EPI, it remains important to investigate whether stage IV BC patients expressed sympathetic nerve fibers and if BC patients are able to respond to the sympathetic stimuli locally in the bone metastasis. To explore this, histological cuts of bone biopsies (comprising both tumor and bone surfaces) retrieved from BC patients (n = 44; Table 2) were submitted to immunofluorescence staining against the rate-limiting catecholamine biosynthesis enzyme (TH—a marker of sympathetic fibers), and the mostly described ADRs in BC (α2a and β2). As can be appreciated in Figure 2, TH-positive nerve fibers were found on the surface of trabecular bone and expressed by tumor cells. Moreover, α2a-expressing cells and β2-positive staining surrounding blood vessels were also detected.

Furthermore, as depicted in Table 2, we were able to identify TH-positive nerve fibers in five patients (out of 44), and cells-expressing TH in 21 patients (out of 44). Furthermore, cells expressing α2a and β2 were found in 18 patients and 43 patients (out of 44), respectively. In addition, we were able to find 13 patients (out of 44) presenting positive staining for TH, α2a, and β2. Nevertheless, no correlations have been established between the presence of sympathetic nerve fibers or ADRs with the clinicopathological data of the BC patients (Table 2). Together, these results suggest that BC patients and the development of metastasis are under the control of SNS activity.

### 3.3. ADR Genes Are Downregulated in BC Tissues and Correlate with a Worse Prognosis

Our data demonstrated that circulating catecholamines (NE and EPI) are elevated in BC patients when compared with healthy donors, with a potential to activate a panoply of nine different adrenoreceptors (α1a, α1b, α1d, α2a, α2b, α2c, β1, β2, and β3) with distinctive physiological outcomes [19].

**Figure 3:**
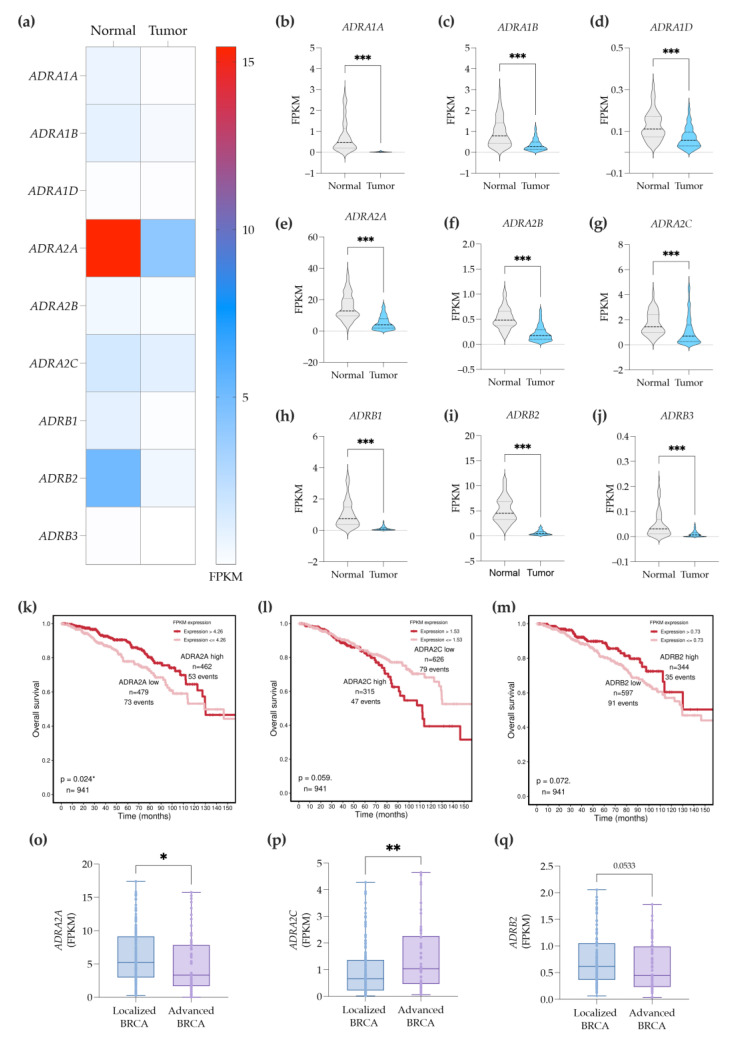
Gene expression profile of ADRs in BC tissues (using the BC dataset included in the TCGA database). (**a**) Heatmap of ADR expression in the TCGA-BC tissues versus their adjacent normal counterparts’ tissues. Charts comparing normal versus BC tissues for each ADR gene expression are illustrated in (**b**–**j**). The survival curves for *ADRA2A* (**k**), *ADRA2C* (**l**), and *ADRB2* (**m**) are depicted. The tumor gene expression of *ADRA2A* (**o**), *ADRA2C* (**p**), and *ADRB2* (**q**) in patients with localized and advanced BC is shown. *p*-value < 0.05 (*); <0.01 (**), <0.001 (***) was considered statistically significant.

In the literature, there are relatively few articles determining the expression of all ADRs in BC tissues. Thus, we further proposed performing a comprehensive evaluation on the expression of all ADRs, using a public and widely explored BC dataset (included in the TCGA database). A broad overview of the ADRs gene expression on normal breast tissue versus BC tissues revealed that the mostly expressed ADR genes (by order of expression) are *ADRA2A*, *ADRB2*, and *ADRA2C* (Figure 3a). Surprisingly, this transcriptomic analysis also demonstrated that the gene expression of all nine distinct ADRs was significantly downregulated in the BC tissues when compared to normal breast tissues (Figure 3b–j). 

The analysis of the survival curves showed that reduced *ADRA2A* gene expression levels in BC tissue was significantly correlated with lower survival rate (Figure 3k) in the BC patients. Inversely, a trend toward increased survival rates in BC was observed at lower expression levels of *ADRA2C* (*p* = 0.06; Figure 3l). Moreover, we also observed that diminished expression levels of the *ADRB2* gene tends to correlate with a lower survival rate (*p* = 0.07; Figure 3m). Overall, these results suggest that the lower expression of the *ADRA2A* gene correlates with the state of the disease.

To further explore this hypothesis, we evaluated the gene expression of *ADRA2A*, *ADRA2C,* and *ADRB2* in patients with localized BC (stage Ia) versus patients with advanced BC, comprising both locally advanced BC (stage IIIc) and metastatic BC (stage IV). Advanced BC patients exhibited decreased expression levels of *ADRA2A* (Figure 3o), while in contrast, the expression of *ADRA2C* was significantly elevated (Figure 3p). A trend toward decreased expression levels of *ADRB2* was also observed (*p* = 0.05; Figure 3q). These results are in accordance with the Kaplan–Meier curves (Figure 3k–m), and suggest that the downregulation of *ADRA2A* and *ADRB2* genes are detrimental for the prognosis of the disease. However, thee *ADRA2C* gene seems to be the exception.

### 3.4. The ADRA2A, ADRA2C and ADRB2 Genes Are Differentially Expressed by the Distinct BC Subtypes

**Figure 4:**
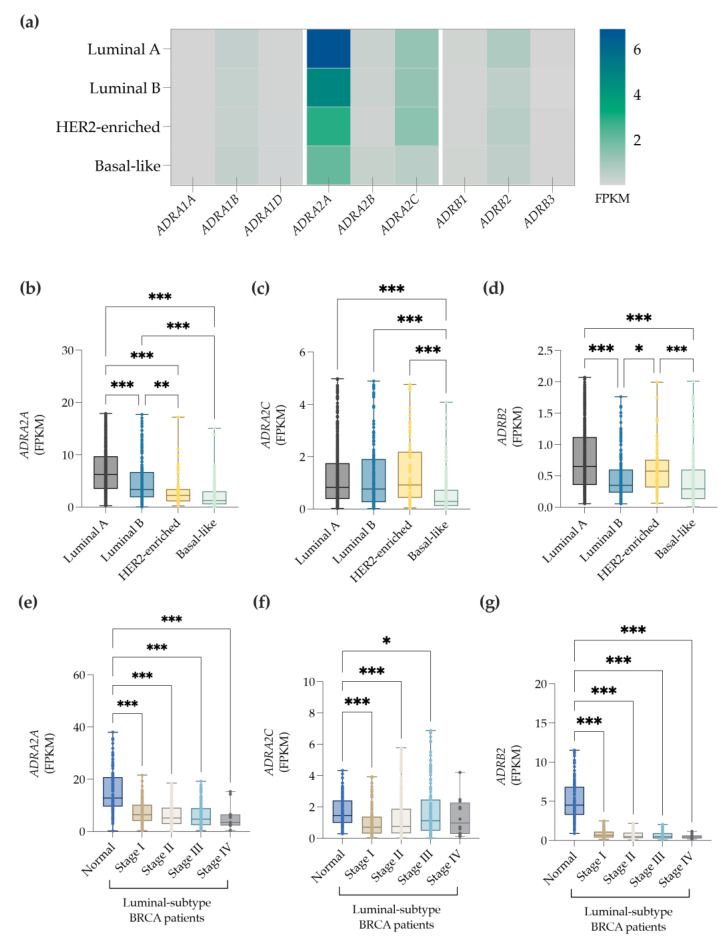
Gene expression profile of ADRs by BC (using the BC dataset included in the TCGA database). (**a**) Heatmap of ADR gene expression in each BC subtype: luminal A, luminal b, HER2-enriched, and basal-like. The tumor gene expression of *ADRA2A* (**b**), *ADRA2C* (**c**), and *ADRB2* (**d**) are represented in column graphs. The gene expression of *ADRA2A* (**e**), *ADRA2C* (**f**), and *ADRB2* (**g**) is shown exclusively in the luminal-subtype patients. *p*-value < 0.05 (*); <0.01 (**), <0.001 (***) was considered statistically significant.

BC subtypes (luminal A, luminal B, HER2-enriched, and basal-like) display different prognosis and disease outcomes [45], with the luminal subtype presenting more propensity to metastasize to the bone [43]. Since 95% of BC patients expressing higher levels of catecholamines are of the luminal subtype, we further characterized the expression profile of each BC subtype. Indeed, we confirmed that the three mostly expressed ADR genes are *ADRA2A*, *ADRA2C*, and *ADRB2* (by order of expression), which are significantly expressed by the different BC subtypes (Figure 4a).

As depicted in Figure 4c, *ADRA2A* is markedly expressed by the luminal A and B subtypes in comparison to the HER2-enriched and basal-like subtypes. Moreover, between the luminal subtypes, *ADRA2A* is mostly expressed by the luminal A subtype (Figure 4b). The transcriptomic analysis also revealed that the *ADRA2C* gene is upregulated in luminal A, luminal B, and Her2-enriched subtypes when compared to the basal-like subtype (Figure 4c). Interestingly, both luminal A and HER2-enriched subtypes express higher levels of the *ADRB2* gene than the luminal B and basal-like subtypes. No differences in the *ADRB2* gene expression were observed between the luminal A and HER2-enriched (Figure 4d). As luminal subtypes generally express higher levels of ADRs than the other BC subtypes, and given its prevalence on bone metastasis [43], we then asked whether the expression of the ADRs in the luminal subtypes was correlated with the progression of the disease. Figure 4e shows that the *ADRA2A* transcripts were significantly decreased in all BC stages (I, II, III, and IV) when compared to normal tissue. Moreover, the expression of *ADRA2C* was also downregulated in stages I, II, and II (Figure 4f), with the exception of stage IV. *ADRB2* expression was also downregulated for all stages of the disease (Figure 4g). These results suggest that the *ADRA2A* and *ADRB2* transcripts are significantly downregulated in luminal-subtype BC patients, and this occurs independently of the stage of the disease.

### 3.5. Markers of Catecholamine Synthesis, Reuptake and Degradation Are also Downregulated in BC Tissues

**Figure 5:**
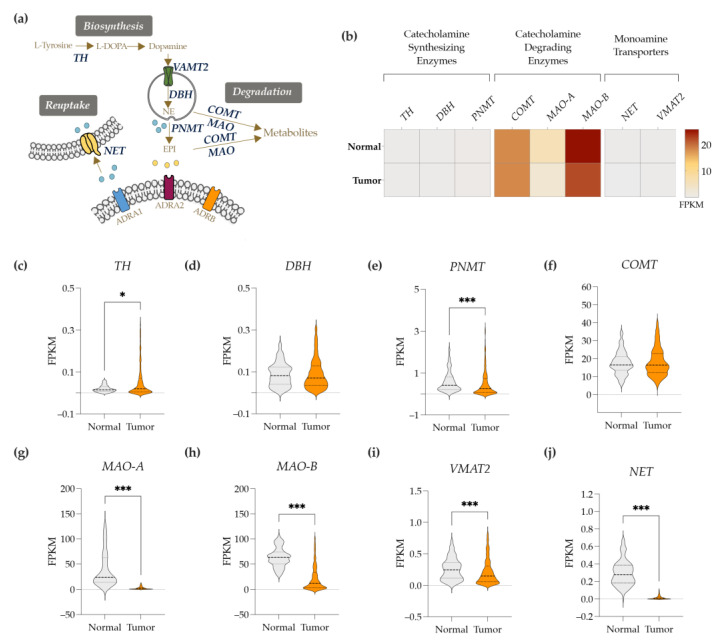
Gene expression profile of enzymes involved in catecholamine biosynthesis, degradation, and transportation in BC (using the BC dataset included in the TCGA database). (**a**) Diagram explaining the metabolism cascade of the catecholamines. (**b**) Heatmap of the expression markers of catecholamine metabolism. (**c**–**j**) Charts comparing normal versus BC tissues for each marker of catecholamine metabolism are illustrated (**b**–**j**). *p*-value < 0.05 (*), <0.001 (***) was considered statistically significant.

Classically, the sources of catecholamine are the adrenal glands (for both NE and EPI) and the terminal nerve fibers (for NE) [46]. However, a great body of evidence has also demonstrated that non-neuronal cells (e.g., immune cells) can express the required machinery to induce the biosynthesis and metabolism of the catecholamines, namely, catecholamine synthesizing (DBH—dopamine beta-hydroxylase; PNMT—phenylethanolamine N-methyltransferase, and TH—tyrosine hydroxylase) and degrading enzymes (COMT—catechol-O-methyltransferase; MAO-A—monoamine oxidase-A; MAO-B—monoamine oxidase-B) as well as monoamine transporters (NET—norepinephrine transporter; and VMAT2—vesicular monoamine transporter 2), as detailed in Figure 5a [47]. Thus, in this order of knowledge, we further explored the expression of such markers on the TCGA-BC dataset. As shown in Figure 5b, both TCGA-BC and their adjacent normal counterpart tissues express higher levels of catecholamine degrading enzymes (e.g., COMT, MAO-A, and MAO-B) in comparison to the catecholamine synthesizing enzymes (TH, DBH, PNMT). Additionally, when comparing normal versus tumor tissues, TH, a rate-limiting enzyme in catecholamine biosynthesis, is upregulated in BC tissues (Figure 5c), although its expression is yet substantially lower in relation to the other markers. Although the DBH gene expression is not different between normal versus tumor tissues, the expression of the PNMT gene is downregulated in BC tissues. As for the expression of the catecholamine degrading enzymes, although no differences were observed on COMT expression, MAO-A and MAO-B transcripts were significantly downregulated in BC when compared to normal tissues. Furthermore, the expression of monoamine transporters such as VMAT2 (responsible for the transport of dopamine to vesicles and therefore NE synthesis) and NET (responsible for the reuptake of local NE) were also downregulated in BC tissues, when compared to normal breast tissues (Figure 5c–j).

We further evaluated possible correlations between ADRs and the expression of the biomarkers of the catecholamine metabolism gene expression levels (Table 3). Regarding the catecholamine biosynthesis, Pearson’s analysis revealed that (i) *ADRA2A* and *ADRB2* were negatively related, while *ADRA1B* and *ADRA2C* were positively correlated with the levels of *TH*; (ii) *ADRA1A*, *ADRA2A*, *ADRA2B*, *ADRB1*, *ADRB2*, *ADRB3* were positively correlated with the levels of *DBH*; and interestingly, (iii) the three mostly expressed ADRs in BC tissue, *ADRA2A*, *ADRA2C,* and *ADRB2*, were positively related with *PNMT* (an enzyme responsible for the synthesis of EPI). Additionally, regarding the catecholamine degradation, our analysis demonstrated that (i) *ADRA1A* and *ADRA2A* were negatively related with *COMT*, while *ADRA2C* was positively related with *COMT*; (ii) *ADRA1A*, *ADRA1D*, *ADRA2A*, *ADRA2C*, *ADRB1*, *ADRB2*, and *ADRB3* were positively related with *MAO-A*; (iii) *ADRA1D*, *ADRA2A*, *ADRB1*, and *ADRB2* were positively related to *MAO-B*. Monoamine transporters are also an important part of catecholamine metabolism [48]. The Pearson’s correlation analysis revealed that (i) *ADRA1A*, *ADRA1D*, *ADRA2B*, *ADRB1*, *ADRB2*, and *ADRB3* were positively related to *NET* expression, while (ii) *ADRA1A*, *ADRA1D*, *ADRA2A*, *ADRA2B*, *ADRB1*, and *ADRB2* were positively related to *VAMT2*.

### 3.6. Human BC Cell Lines Express Higher Levels of ADRA2C and COMT Genes

**Figure 6:**
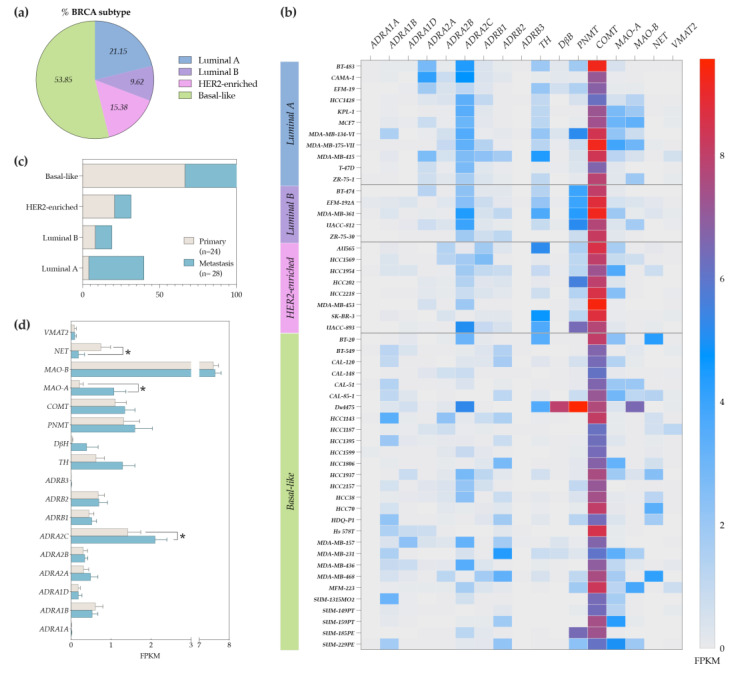
Adrenergic profile of human BC cell lines (dataset obtained from CCLE database). (**a**) Percentage of human BC cell line subtypes. (**b**) Heatmap depicting the gene expression of sympathetic markers for all human BC cells lines analyzed and categorized by each BC subtype. (**c**) Human BC cell lines subdivided in primary versus metastasis cell lines. (**d**) Gene expression of sympathetic markers on primary versus metastasis of human BC cell lines. *p*-value < 0.05 (*) was considered statistically significant.

BC cell lines have been widely used as feasible models to study BC and its progression to BC metastasis. As a proof of concept, to confirm our previous data, we aimed to perform a complete characterization on the adrenergic profile of BC cell lines. To accomplish this, a dataset comprising several BC cell lines was selected from a publicly available database (obtained from CCLE dataset). For this analysis, we excluded cell lines lacking all the information needed as well as the cell lines whose status of primary markers and BC subtype is still not clearly identified [42], resulting in a final number of 52 human BC cell lines analyzed. From all of the BC cell lines analyzed, 54% represent basal-like, 21% luminal A, 10% luminal B and 15% HER2-enriched BC subtypes (Figure 6a). As can be appreciated in Figure 6b, the transcriptomic analysis on the expression of sympathetic markers uncovered the sympathetic profile on BC cell lines. In fact, this analysis revealed that *ADRA2C* transcripts are highly expressed by most cell lines, particularly by the luminal A, luminal B, and HER2-enriched subtypes (Figure 6b). *ADRA2A* and *ADRB2* genes are also expressed by the BC cell lines, though in inferior levels. Additionally, catecholamine synthesizing enzymes (*TH*, *DBH,* and *PNMT*) as well as the catecholamine degrading enzyme *COMT* are highly expressed in both the luminal and HER2-enriched subtypes or by all BC subtypes, respectively. Overall, each BC cell line is unique and exhibits a distinctive adrenergic profile. With the exception of the BC cell line, MDA-MB-415, this profile differs greatly from the gene expression profile observed in human cohort from the BC-TCGA database (Figure 3, Figure 4 and Figure 5), which should be taken into consideration in future work.

BC cells are retrieved from individual BC patients at different stages of the disease. Hence, in accordance with the sample collection site, BC cell lines can be classified as primary (collected within breast tissue) or metastasis (collected outside the breast, e.g., skin, pleural effusion, ascites, lymph nodes, pericardial effusion, central nervous system). In line with the scope of this study and given the relevance of the sympathetic signature in BC and the establishment of metastasis [45], we further pursued studies evaluating whether the sympathetic marker genes were differentially expressed between the primary (n = 24) versus metastatic BC cell lines (n = 28; Figure 6c,d). As shown in Figure 6c, most primary BC cell lines represent the basal-like subtype, while the metastatic BC cell lines were distributed along the four different subtypes (luminal A, luminal B, HER2-enriched, and basal-like). Interestingly, our analysis indicated that among the sympathetic markers, the *ADRA2C* and *MAO-A* genes were significantly upregulated, while the *NET* gene was significantly downregulated by the metastatic BC cell lines when compared to the primary BC cell lines (Figure 6d). These results highlight the functional role of the α2c-ADR signaling pathway on metastatic BC and opens up the possibility that metastatic BC cells might be able to modulate the uptake and degradation of catecholamines locally.

## 4. Discussion

Previous reports support the hypothesis that chronic SNS activation plays a critical role in BC and the development of metastasis. These indications have mainly been validated in preclinical models using immunocompromised mice [13,15,34], and the information gathered regarding the role of SNS activity in human BC samples is scarce and occasionally contradictory [11]. Herein, we found evidence that BC patients are indeed under the control of SNS activity, as the catecholamines (NE and EPI) in circulation released by the adrenal glands are augmented when compared with healthy donors. This is more preeminent at advanced stages of the disease, specifically in BC patients with bone metastasis that exhibited elevated levels of circulating EPI in opposition to the BC patients without metastasis. A comprehensive analysis using the BC-TCGA dataset obtained from the TCGA database revealed that the ADRs (*ADRA2A*, *ADRA2C,* and *ADRB2*) as well as the catecholamine synthesizing (*PNMT*) and degrading enzyme (*MAO-A* and *MAO-B*) genes are downregulated in BC tissues. Importantly, the lower expression of ADRs such as the *ADRA2A*, *ADRA2C*, and *ADRB2* genes, is correlated with metastatic BC and thus, the prognosis of the disease.

BC patients at a given time of the disease, either at the time of the diagnosis, surgery, or treatment, are at higher risk of feeling emotional stress and developing anxiety and depression disorders [49,50,51,52,53]. Our observations not only confirmed that the catecholamines (NE and EPI) in circulation are elevated in BC patients, but that this increase is associated with the development of distant metastasis, specifically in the bone microenvironment. Previous pharmaco-epidemiological studies in BC patients have suggested that the use of β-blockers has a protective effect on the progression of the disease and the development of metastasis [54,55,56,57,58], increasing patient survival. However, this beneficial effect has not been replicated in some epidemiologic studies [59,60]. Recent studies have reported that the association between the use of β-blocker and prolonged BC survival is subtype-specific to triple-negative BC patients [61,62], which might explain the contradictory data. Overall, our results strongly suggest the therapeutic potential of targeting the SNS as an adjuvant or neoadjuvant therapy for BC disease. This approach is already being explored in two clinical trials using propranolol (a widely used β-signaling blocker in the clinic), however, these studies did not deliver conclusive results (NCT01847001; NCT02596867). Since clinical studies are still scarce, the role of β receptors in BC progression and the beneficial usage of β-blockers are still a matter of debate that requires more investigation.

In light of the results described herein, we are now able to hypothesize that, aside from β2, other adrenergic signaling pathways are strong players in this dynamic “game” between SNS and BC, namely the α2a- and α2c-pathways. The profiling performed on the human BC tissues and human BC cells lines revealed that α2a, α2c, and β2 are the ADRs mostly expressed. In addition, we demonstrated that the *ADRA2A* gene is highly expressed in the luminal A and B subtypes, the BC subtypes with a higher propensity to metastasize to the bone [43]. Thus, apart from the known broad expression of β2, we also confirmed the presence of α2a in bone biopsies. The expression of *ADRA2A*, *ADRA2C,* and *ADRB2* genes in BC tissues has previously been explored [63], specifically to unveil the association between ADRs and the clinical outcome. However, this is the first time that a complete characterization and profiling on the adrenergic system has been performed.

Surprisingly, the comprehensive analysis performed using the TCGA database revealed that all ADR genes were downregulated in BC patients. This is particularly interestingly since we observed a significant increase in the circulating levels of NE and EPI in the BC patients. ADR belongs to the wide-ranging G-protein coupled receptor (GPCR) family. This specific family of receptors has been extensively shown to be internalized and degraded upon stimulation, and thus its expression is downregulated through specific signaling mechanisms [64]. Furthermore, as suggested in other pathologies (e.g., heart failure), the downregulation of ADR might occur due to overstimulation of the ADRs due to higher plasma levels of NE and EPI, negatively dictating the progression of the disease [64]. Moreover, in contrast to the ADRs and other catecholamine synthesizing enzymes, we also observed a slight upregulation on the *TH* gene in BC tissues, suggesting that there is an increase in the NE released locally in the BC tissues by the sympathetic nerve fibers. This observation is in line with a previous report showing that there is a sprouting of TH-nerve fibers in BC [12]. Together, our results indicate that long term ADR-stimulation might negatively impact the development of BC.

Another interesting aspect related to catecholamine metabolism rises from the fact that we have also observed a significant downregulation of the catecholamine degrading enzymes genes, *MAO-A* and *MAO-B*, and the NE transporter gene, *NET* in BC tissues. This evidence suggests that BC tissues lose their capacity to metabolize local catecholamines and to reuptake excessive levels of local NE. Thus, this supports our previous hypothesis that there is an agonist-overstimulation in BC tissues, leading to the downregulation of ADR genes and the associated signaling pathways. Nevertheless, the local levels of NE released by the sympathetic nerves within breast/tumor tissue as well as the amount of catecholamines (both NE and EPI) that reach the BC tissue remain unknown. Growing evidence suggests that the local release of NE from the SNS nerve terminals is in fact the dominant driving force in the sympathetic control of cancer progression [65,66,67]. Future work should be performed to evaluate the levels of catecholamines released locally by the nerve terminals within human BC tissue and in bone metastasis.

The use of BC cell lines in the field of BC research has been extensively described in both in vitro and in vivo studies. For this matter, MCF-7 and MDA-MB-231 are the cell lines mostly employed [24,68]. Nonetheless, as discussed elsewhere [45], the results obtained in previous work regarding the physiological role of stress in BC development are often contradictory and sometimes difficult to replicate, in particular, the use of β-blockers in BC patients. Our comprehensive analysis on the adrenergic profile of human BC cell lines using the CCLE database showed for the first time that the genes of ADRs, catecholamine metabolic enzymes, and transporters were differently expressed depending on the BC cell line and BC subtype, and thus might justify this problem. Most importantly, given our results, researchers can now explore an individual question/hypothesis by selecting a specific human cell line from a list of 52 BC cell lines, which comprises the fundamental adrenergic characteristics (as such α2c-, β2- or TH-expressing cell lines, etc.) to conduct their experiments.

We have to acknowledge a few study limitations, which should be addressed in future studies. First, we did not observe statistical differences on CORT levels (another stressor marker). This might be explained by the fact that the patients’ sample collection was performed over a wide time window (8 am to 3 pm). This is specifically critical since CORT levels alter along the day and its release peak varies from patient to patient. The amount of CORT in circulation would be better evaluated when collected over a period of 24 h in urine samples. Second, regarding the human bone biopsies used in this study to evaluate the presence of sympathetic nerve fibers and ADRs, the sample size as well as the location of collection were widely variable, which hindered a quantitative analyses.

## 5. Conclusions

In summary, it is clear that the SNS might affect the development of BC, since BC patients exhibited increased levels of circulating catecholamines (NE and/or EPI) at all stages of the disease. These neuromediators, NE and EPI, are capable of physiologically activating nine different ADRs (α1a, α1b, α1d, α2a, α2b, α2c, β1, β2, and β3). Interestingly, BC patients displayed worse prognosis when the gene expression of such ADRs was deregulated. In light of our comprehensive study, we were able to demonstrate that there were striking correlations between the expression of ADR genes (e.g., *ADRA2A*, *ADRA2C*, and *ADRB2*) and the prognosis of the disease and the development of metastasis. Moreover, β2-ADR is not the most strongly expressed receptor in normal breast tissues, neither in BC tissues. This was evident when studying both the human BC tissues and human BC cell lines.

Indeed, previous epidemiological and functional in vivo and in vitro studies on the role of adrenergic signaling in BC have been focused on a particular adrenergic pathway, the β2-signaling pathway. Therefore, our results might explain the controversy and the lack of clinical results when using β-blockers as an adjuvant/neoadjuvant therapy for BC disease.

Overall, there are questions that remain to be addressed in future studies to (i) clarify the protein expression and tissue distribution of ADRs in primary tumors versus metastases in a comprehensive cohort of BC patients, comprising all BC subtypes; (ii) investigate the mechanisms of receptor desensitization/downregulation in the context of BC microenvironment; and finally, (iii) evaluate the possible synergetic actions between the α2- and β2-signaling pathways in BC either in in vitro or in vivo settings.

## Figures and Tables

**Table 1:** Detailed clinicopathological data of the BC patients and healthy controls.

	Healthy Donors	BC Patients
Patients (n)	18	72
Median age at diagnosis *(min–max)*	57 (46–65)	59 (30–93)
Median tumor size (mm) *(min–max)*	−	26 (0.3–67)
Molecular subtype * (%)		
*Luminal A*	−	39 (54.2)
*Luminal B*	−	29 (40.3)
*HER2-enriched*	−	2 (2.8)
*Basal-like/TNBC*	−	2 (2.8)
Histological type (%)		
*Invasive ductal carcinoma*	−	53 (73.6)
*Invasive lobular carcinoma*	−	14 (19.4)
*Mixed-type carcinoma*	−	2 (2.8)
*Special subtype*	−	3 (4.2)
Grade (%)		
*G1*	−	1 (1.4)
*G2*	−	42 (58.3)
*G3*	−	28 (38.9)
*Gx*	−	1 (1.4)
T stage (%)		
*T1*	−	31 (43.1)
*T2*	−	19 (26.4)
*T3*	−	14 (19.4)
*T4*	−	8 (11.1)
N stage (%)		
*N0*	−	32 (44.4)
*N1*	−	15 (20.8)
*N2*	−	8 (11.1)
*N3*	−	8 (11.1)
*Nx*	−	9 (12.5)
M stage (%)		
*M0*	−	51 (70.8)
*M1*	−	18 (25.0)
*Mx*	−	3 (4.2)
Menopause Status (%)		
*Pre*	−	16.7
*Post*	−	83.3

* As determined by immunohistochemistry analysis. Abbreviations: HER2—human epidermal growth factor receptor 2; TBNC—triple-negative breast cancer.

**Table 2:** Correlations between the clinicopathological data of the BC patients and the expression of a sympathetic innervation marker (TH) and adrenergic receptors (α2a and β2) in bone metastasis biopsies retrieved from BC patients.

	Total	TH+	α2a+	β2+	TH/α2a/β2+
**Patients (n)**	44	21	18	43	13
Mean age at diagnosis *(min-max)*	52 (28–76)	52 (28–76)	51 (32–73)	51 (28–76)	51 (32–73)
Tumor size (mm) *(min-max)*	30 (9–100)	33 (10–100)	32 (9–100)	30 (9–100)	37 (15–100)
Molecular subtype * (%)					
*Luminal A*	32	24	39	33	38
*Luminal B*	66	76	56	65	62
*HER2-enriched*	0	0	0	0	0
*Basal-like/TBNC*	2	0	5	2	0
Grade (%)					
*G1*	6.8	4.8	5.6	7.0	0
*G2*	52.3	52.4	50.0	53.5	53.8
*G3*	40.9	42.8	44.4	39.5	46.2

* As determined by the immunohistochemistry analysis. Abbreviations: HER2—human epidermal growth factor receptor 2; TBNC—triple-negative breast cancer; TH—tyrosine hydroxylase.

**Table 3:** Pearson’s correlation between the gene expression of ADRs and markers of catecholamine metabolism in the BC tissues (dataset obtained from TCGA database).

	*ADRA1A*	*ADRA1B*	*ADRA1D*	*ADRA2A*	*ADRA2B*	*ADRA2C*	*ADRB1*	*ADRB2*	*ADRB3*
***TH***									
Pearson’s correlation	−0.012	0.072 *	0.033	−0.091 **	0.002	0.127 ***	0.015	−0.028	0.002
*p-*value	0.736	0.047	0.360	0.010	0.961	0.000	0.692	0.438	0.966
***DBH***									
Pearson’s correlation	0.126 ***	−0.008	−0.008	0.087 *	0.190 ***	0.068	0.136 ***	0.249 ***	0.155 ***
*p-*value	0.000	0.816	0.816	0.012	0.000	0.056	0.000	0.000	0.000
***PNMT***									
Pearson’s correlation	0.007	0.020	0.020	0.168 ***	0.040	0.166 ***	0.042	0.136 ***	0.056
*p-*value	0.845	0.595	0.595	0.000	0.280	0.000	0.272	0.000	0.129
***COMT***									
Pearson’s correlation	3.4925 ***	2.070 *	1.321	−2.463 *	4.163	5.470 ***	−0.979	−1.161	0.897
*p-*value	0.000	0.039	0.187	0.014	0.000	0.000	0.328	0.246	0.370
***MAO-A***									
Pearson’s correlation	7.426 ***	0.595	2.605 **	7.647 ***	0.978	2.227 *	6.060 ***	10.056 ***	5.329 ***
*p-*value	0.000	0.5519	0.009	0.000	0.328	0.026	0.000	0.000	0.000
***MAO-B***									
Pearson’s correlation	0.415	1.901	4.271 ***	7.300 ***	1.809	1.596	2.323 *	7.185 ***	0.705
*p-*value	0.677	0.058	0.000	0.000	0.071	0.111	0.020	0.000	0.481
***NET***									
Pearson’s correlation	4.595 ***	1.085	5.750 ***	1.348	7.091 ***	1.138	5.685 ***	8.675 ***	5.856 ***
*p-*value	0.000	0.278	0.000	0.178	0.000	0.255	0.000	0.000	0.000
***VMAT2***									
Pearson’s correlation	5.338 ***	−0.196	3.541 ***	10.442 ***	−2.013 *	1.236	2.070 *	10.345 ***	1.058
*p-*value	0.000	0.884	0.000	0.000	0.044	0.217	0.039	0.000	0.290

*p*-value < 0.05 (*), <0.01 (**), <0.001 (***) were considered statistically significant. Abbreviations: ADRA—adrenoreceptor alpha; ADRB—adrenoreceptor beta; COMT—catechol-O-methyltransferase; DBH—dopamine beta-hydroxylase; MAO-A—monoamine oxidase-A; MAO-B—monoamine oxidase-B; NET—norepinephrine transporter; PNMT—phenylethanolamine N-methyltransferase; TH—tyrosine hydroxylase; VMAT2—vesicular monoamine transporter 2.

## Data Availability

All data generated or analyzed during this study were included in this article. The datasets used and/or analyzed in this study are available from the corresponding author upon reasonable request.
